# A Scoping Review of Footwear Worn by People With Diabetes in Low- and Middle-Income Countries: Implications for Ulcer Prevention Programs

**DOI:** 10.9745/GHSP-D-22-00392

**Published:** 2023-04-28

**Authors:** Madison Reddie, Christopher Shallal, Daniel Frey

**Affiliations:** a Department of Mechanical Engineering, Massachusetts Institute of Technology, Cambridge, MA, USA.; b Harvard University-Massachusetts Institute of Technology Health Sciences and Technology, Cambridge, MA, USA.

## Abstract

International guidelines advise people with diabetes to wear close-toed, thick-soled footwear to protect against foot ulceration. However, this type of footwear is incompatible with some of the cultures, climates, and socioeconomic conditions in many low- and middle-income countries (LMICs). This scoping review aims to summarize what is known about footwear used by people with diabetes in LMICs and consider whether international diabetic foot guidelines are practical and actionable in these contexts, given current practices. PubMed, CINAHL, Scopus, Embase, Web of Science, Latin American and Caribbean Health Sciences Literature, and African Journals Online were searched for articles that documented the footwear used by people with diabetes in LMICs. Twenty-five studies from 13 countries were eligible for inclusion and indicated that a large proportion of people with diabetes wear footwear that is considered inappropriate by current guidance, with sandals and flip-flops being popular choices in a majority of the studies. Reasons given for these choices include poverty, lack of awareness of and provider communication about the importance of footwear selection, comfort, and cultural norms. We recommend that LMIC health care systems relying on international guidelines critically consider whether their recommendations are sensible in their settings. Diabetic foot experts and LMIC-based health care stakeholders should collaborate to design alternative guidelines, strategies, and interventions specifically for LMIC contexts to increase preventative practice feasibility and uptake.

## Abstract (precis)

New approaches to reducing the massive burden of diabetic foot ulceration are needed for low- and middle-income country settings, where current international guidelines may not be practical.

## Teaser

**Key Findings:**

- Few people with diabetes in low- and middle-income countries wear footwear recommended by international guidelines for ulcer prevention.
- Poverty, culture, climate, health literacy, and the nature of health care in low- and middle-income countries are barriers to implementation of international ulcer prevention guidelines.

**Key Implication:**

- Public health officials and clinicians in low- and middle-income countries should consider whether they are well served by current international diabetic foot ulcer prevention guidelines and work with diabetic foot experts to develop more practical strategies and programs for low-resource contexts.

## INTRODUCTION

The International Diabetes Federation estimated in 2021 that at least 537 million adults, or 10.5%, have diabetes, more than 3 times as many as in 2000. Diabetes prevalence and incidence rates continue to increase due to globalization, urbanization, and associated lifestyle changes. Low- and middle-income countries (LMICs), where these trends have been especially pronounced in the 21st century, are now home to more than 80% of people with diabetes globally. Furthermore, 94% of the new diagnoses between 2021 and 2045 are predicted to be in LMICs.^1^

The costliest complications of diabetes are those affecting the lower extremities, accounting for roughly a third of all spending on diabetes treatment.^2^ Impaired circulation, motor function, and sensation in the feet make people with diabetes vulnerable to diabetic foot ulcers (DFUs). Among people with diabetes,^3^ DFUs are the most common reason for hospitalization and are responsible for 61% of the years lived with disability, putting DFUs conservatively among the top 10 conditions causing disability worldwide.^4^ The mortality rate for people with DFUs is nearly identical to that of cancer.^2^ Thus, DFU prevention is a public health priority, but it is hindered by suboptimal patient self-care behaviors (e.g., poor footwear practice).^5^

Between 19%–34% of people with diabetes will develop a DFU in their lifetime,^6^ and at least 1 million people with diabetes have a lower extremity amputated every year,^7^ causing extreme financial, physical, social, and emotional distress,^8^^,^^9^ and significantly increasing risk of mortality.^3^^,^^10^^,^^11^ Low rates of access to quality prosthetics, accessible infrastructure, and physical and occupational therapy in LMICs leave many amputees immobile and prevent them from ever recovering in all of these respects.^12^^,^^13^

Encouragingly, 85% of diabetic lower-extremity amputations are preceded by DFUs, of which the etiology and risk factors are fairly well understood. Therefore, these amputations are considered largely preventable.^7^^,^^14^^–^^16^ High pressure from unsupportive or ill-fitting shoes and injuries sustained while not wearing protective footwear are among the most commonly identified causes of DFUs, making good footwear choices a critical pillar of DFU prevention.^8^^,^^15^^,^^17^^–^^19^ Hence, “appropriate” or “proper” footwear has been recommended for people with diabetes in published clinical practice guidelines and the medical literature for at least 15 years^20^ and in international consensus documents for over 10 years.^15^^,^^21^^,^^22^ The international guidelines consider footwear “appropriate” for people with diabetes if it is closed, is sufficiently long and wide, has a thick sole with a soft insole and hard outer sole, effectively distributes pressure on the bottoms of the feet, and contains no seams or other protruding features inside. Special or custom therapeutic footwear with features beyond these is recommended for those at high risk of DFU. These guidelines are authored primarily by experts in high-income countries (HICs) and based on research conducted in those regions; whether they currently translate into practice in LMICs has yet to be comprehensively investigated.

High pressure from unsupportive or ill-fitting shoes is among the most commonly identified causes of DFUs, making good footwear choices a critical pillar of DFU prevention.

A few researchers and clinicians in LMICs have expressed frustration at the neglected difficulty of implementing or compelling patients to comply with the guidelines. Jain and Apoorva^23^ wrote:

*… when it comes to developing countries like India, myriad factors combine to render a fixed protocol/guidance on footwear unsustainable. Socioeconomic conditions, cultural factors, beliefs, religious factors and attitudes, for example, all play a vital role in influencing footwear practices in India and other developing countries.*

Similarly, Isip et al.^24^ from the Philippines pointed out that international diabetic footwear recommendations are “made for countries with cooler climates and good podiatry services” and are “not logistically feasible in our setting.” They voiced disappointment at the lack of guidance and public health programs for helping high-risk patients to select sensible alternatives to custom therapeutic shoes, which they do not have access to.

Open-toed footwear that is popular and considered comfortable in many LMICs, especially those with tropical climates, is potentially dangerous to people with diabetes, but the recommended footwear may be contextually incompatible with these settings. While the International Working Group on the Diabetic Foot’s 2015 footwear guidance^22^ briefly acknowledged the nonuniversal applicability of their guidelines and the need for alternative public health strategies in LMICs, there is no progress reported in their 2019 update.^15^ The issue does not appear in their research agenda. The only alternative recommendation offered for low-resource settings is^15^:

*… where this [therapeutic footwear and accurate technology for pressure measurement] cannot yet be accommodated, we suggest to prescribe therapeutic footwear using available state-of-the-art scientific knowledge on footwear designs that effectively offload the foot.*

This recommendation is still far out of reach for many in LMICs.

While the literature on adherence to prescription footwear among people with diabetes, at least in the HICs, is growing,^25^^–^^30^ far less is known about whether people with diabetes without access to therapeutic footwear adhere to recommendations to wear protective shoes.^15^ The footwear practices of people with diabetes are especially of public health interest in LMIC contexts as, in general, common footwear tends to be less protective than in HICs. Furthermore, advanced podiatric, limb-salvage, and rehabilitation services are less accessible to those who do develop DFUs in LMICs, and delays in care-seeking are common.^12^^,^^31^ These additional challenges make primary prevention all the more critical.

To the best of our knowledge, this topic has never been reviewed. The only footwear practices discussed in the 2021 scoping review of global diabetic foot self-care knowledge and habits by Manickum et al.^5^ are barefoot walking, footwear inspection, and therapeutic footwear use. In some of the studies included in their review, no participants reported using therapeutic shoes. But specialized footwear is not recommended for all people with diabetes,^15^ and what types of footwear were actually used and why were not presented, leaving an important knowledge gap. To develop public health strategies for DFU prevention for LMICs, current conditions and the factors that influence them must be understood.

To develop public health strategies for DFU prevention for LMICs, current conditions and the factors that influence them must be understood.

The available data regarding the footwear used by people with diabetes in LMICs come from studies with diverse methods and objectives, and the data itself are recorded and reported heterogeneously. However, these data are of great importance for determining whether available evidence suggests a need for alternative guidelines and public health programs for low-resource settings, as some researchers propose based on their experiences.^23^^,^^24^ These data will also inform global diabetic foot experts and public health practitioners about the context in which such guidance or programs must fit. Published evidence regarding footwear practice among people with diabetes in LMICs is thus of significant international interest but is not compatible with systematic review or meta-analysis techniques, suggesting the value of a scoping review.^32^^,^^33^

Preliminary literature searches informed the following research question, formulated using the PCC (Population, Concept, Context) format^34^: What is known about the footwear used by people with diabetes in low- and middle-income countries? Guided by this research question, the objectives of this scoping review are (1) to investigate the comprehensiveness of available data describing the footwear worn by people with diabetes in LMICs, (2) to collect and disseminate the identified data, and (3) to consider what the current state of knowledge indicates about the feasibility of current footwear guidelines for people with diabetes in LMIC contexts and how it can inform public health practice.

## METHODS

The scoping review methodology was developed before the formal literature search based on the methodological framework for scoping reviews laid out by Arksey and O’Malley^32^ and elaborated by Levac et al.^35^ and Peters et al.,^33^ in consultation with our institution’s biosciences librarian, and using the Joanna Briggs Institute (JBI) guidance on scoping reviews.^34^ Reporting is also done in accordance with the PRISMA extension for scoping reviews.^36^ The methodology follows the 5 steps outlined by Arksey and O’Malley^32^: identifying the research question; identifying relevant studies; study selection; charting the data; and collating, summarizing, and reporting the results.

### Search Strategy

The search strategy was iteratively developed in consultation with our institution’s biosciences librarian. Publications from 2010 to the present were sought through a 4-step search strategy, with 1 step added to the JBI’s recommended 3 steps.^34^ Initial informal searches were conducted by the first author on PubMed and Google Scholar to identify relevant sources. Roughly 20 results that discussed the footwear worn by people with diabetes in LMICs were analyzed to inform the search. Search terms were drawn from text language, index terms, and keywords of these articles related to the research question. Second, we consulted with our institution’s biosciences librarian and iteratively tested various combinations of search terms and limits in PubMed, seeking to keep the search as broad as possible and incorporating feedback from the librarian until a manageable number of relevant results were returned. Search relevance was gauged using the 20 previously identified relevant documents and other search result titles.

Third, in December 2021, searches of PubMed, CINAHL, Scopus, Embase, Web of Science, Latin American and Caribbean Health Sciences Literature, and African Journals Online were conducted by the first author. Search terms included diabetes, diabetic, footwear, foot wear, shoes, worn, wear, preference, practice, habits, and related variations of these words. Search queries were adapted to each database as appropriate. Searches were conducted in English, but no restriction was imposed on language. The PubMed search query is available in the Supplement. In databases that accommodated it, sources from HICs, ineligible publication types, nonhuman studies, and pediatric studies were filtered out. All electronic database search results were exported to EndNote, and duplicates were removed using EndNote’s duplicate identification function. As a particularly relevant source, the complete online archive of the Diabetic Foot Journal was also manually searched by the first author in December 2021. As the fourth step, after full-text screening, references of included articles were screened by the first author for additional relevant articles. Eligible articles previously known to the authors that did not appear in the prior steps were included. The references of the related scoping review by Manickum et al.^5^ were also screened.

### Inclusion Criteria

Peer-reviewed, published primary research studies of any study design (except for case reports), quantitative or qualitative, were eligible for inclusion. We included only published literature because we sought data that were systematically collected from a sample from the population of interest as opposed to anecdotal information that may have been more subject to bias. Hence, commentaries, opinions, letters to editors, abstracts, protocols, and gray literature were not included. Reviews were not excluded from the search but were sought principally to identify primary research studies that were eligible for inclusion. As noted previously, no existing reviews of footwear used by people with diabetes in LMICs were found.

Studies of people with type 1 and/or type 2 diabetes aged older than 18 years and residing in 1 or more LMICs (per World Bank income-level classifications^37^) were eligible for inclusion. Where minimum participant age was not explicitly stated and it was not indicated that there were any pediatric participants, samples were assumed to be aged older than 18 years. Where participant diabetes types were not explicitly stated, types 1 and 2 only were assumed to be present in the sample. We limited eligible studies to those published from 2010 onward to reflect reasonably current economic conditions and consensus on appropriate footwear for people with diabetes. We included studies that reported the type of footwear preferred or worn by at least 50% of the study sample, with this information either self-reported by participants or observed by researchers. We excluded studies including participants aged younger than 18 years, including only participants with prescribed footwear or providing participants with therapeutic footwear, reporting only the fit and not the type of footwear, asking participants only whether their footwear was subjectively “comfortable” or using unclear footwear classification schemes, reporting knowledge about appropriate footwear but not footwear practice, and of a case study design.

### Study Selection

Two reviewers independently screened titles and abstracts in Endnote using a list of inclusion/exclusion criteria. Two independent reviewers (1 who also screened titles and abstracts and 1 who did not) conducted full-text screenings. Necessary full texts were obtained through the authors’ institution’s holdings and interlibrary loan service. Despite requests through interlibrary loans and seeking author contact information, 2 full texts could not be retrieved.^38^^,^^39^ Sources published in a language other than English were translated to English using Google’s document translation tool. Reviewers used a standardized, closed-ended checklist of the inclusion/exclusion criteria in Microsoft Excel during full-text screening to confirm source eligibility. The inclusion/exclusion form was developed by the first author and then pilot tested by 2 reviewers using 10 randomly selected English language articles that were eligible for full-text screening. The pilot test resulted in 90% agreement, and discrepancies were effectively resolved through discussion. Specific reasons for exclusion for each excluded full text were recorded on the form.

Disagreements between the 2 reviewers at both the title/abstract and full-text screening stages were resolved by discussion and, if necessary, after discussion, consultation with a third reviewer.^33^^–^^35^ Studies identified through citation searching and manual searching were screened by only the first author due to the large number of sources.

### Charting the Data

A standard, open-ended data charting form was developed by the first author in Excel before data extraction. Two reviewers pilot tested the form independently using 7 eligible articles known to the authors before the literature search. The pilot test demonstrated that the form facilitated consistent and complete information capture. The first author charted the data for all included articles, recording the study authors, publication year, location, design, objective, number of subjects, important sample characteristics, and results relevant to subjects’ footwear on the form. Sample characteristics were considered important if the reviewer believed that they could affect footwear choice (e.g., gender, education, income, occupation, geography, duration/severity of disease, diabetic foot symptoms, DFU history, and health care habits). Characteristics of interest were not defined in advance because the reviewers could not anticipate every variable that might be reported. A third reviewer checked the completed data extraction form for unexpected or inconsistent entries and completion.

### Collating, Summarizing, and Reporting Results

The charted data are reproduced in figures and tables, and quantitative and qualitative findings are presented. For data given in absolute numbers as opposed to percentages of the study population, percentages were calculated to simplify reporting. All numerical values are reported to 2 significant digits for simplicity. The heterogeneous nature of the data precludes a meta-analysis, but general trends and notable patterns related to geographies, various sample characteristics (e.g., education level), and time were searched for by critically examining the data chart.

Unlike a systematic review, the purpose of this review is not to make recommendations for clinical practice nor to evaluate the effectiveness of an intervention but rather to characterize and summarize the evidence landscape on a topic for the first time. Hence, a risk-of-bias assessment is not conducted for included studies, consistent with the definition of scoping reviews and current methodology recommendations from the JBI and others.^32^^–^^34^^,^^40^

## RESULTS

We describe the results of the literature search and screening process, characterize the studies meeting the inclusion criteria, present the studies’ findings related to footwear practices, and discuss factors found to be affecting those practices.

### Search Results and Screening

**FIGURE 1:**
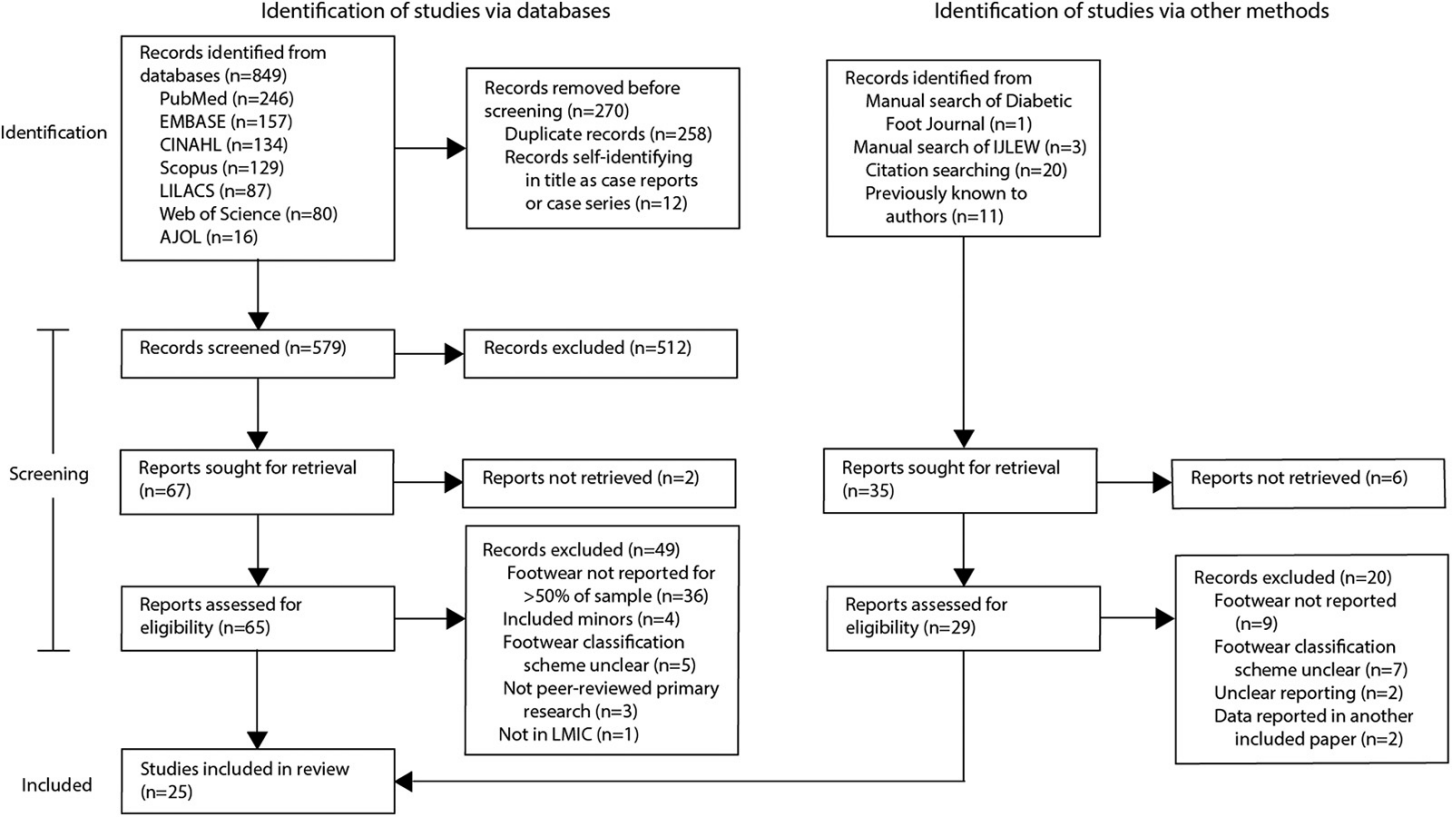
Screening Process for Review of Footwear Worn by People With Diabetes In Low- and Middle-Income Countries Abbreviations: AJOL, African Journals Online; CINAHL; Cumulated Index to Nursing and Allied Health Literature; IJLEW, International Journal of Lower Extremity Wounds; LILACS, Latin American and Caribbean Health Sciences Literature; LMIC, low- and middle-income country.

Figure 1 displays the number of records identified by the database searches and other search processes, the number of records excluded at each screening stage, reasons for their exclusion, and the final number of included articles. The database search returned 849 records, from which 258 duplicates and 12 case reports were removed, leaving 579 records screened by title and abstract. We retrieved 67 full texts from the search and another 35 records identified from the manual searches and citation searching or previously known to the authors. After full-text screening, 25 articles were found to meet the inclusion criteria and are included in the review.^24^^,^^41^^–^^64^

### Characteristics of Included Studies

**FIGURE 2:**
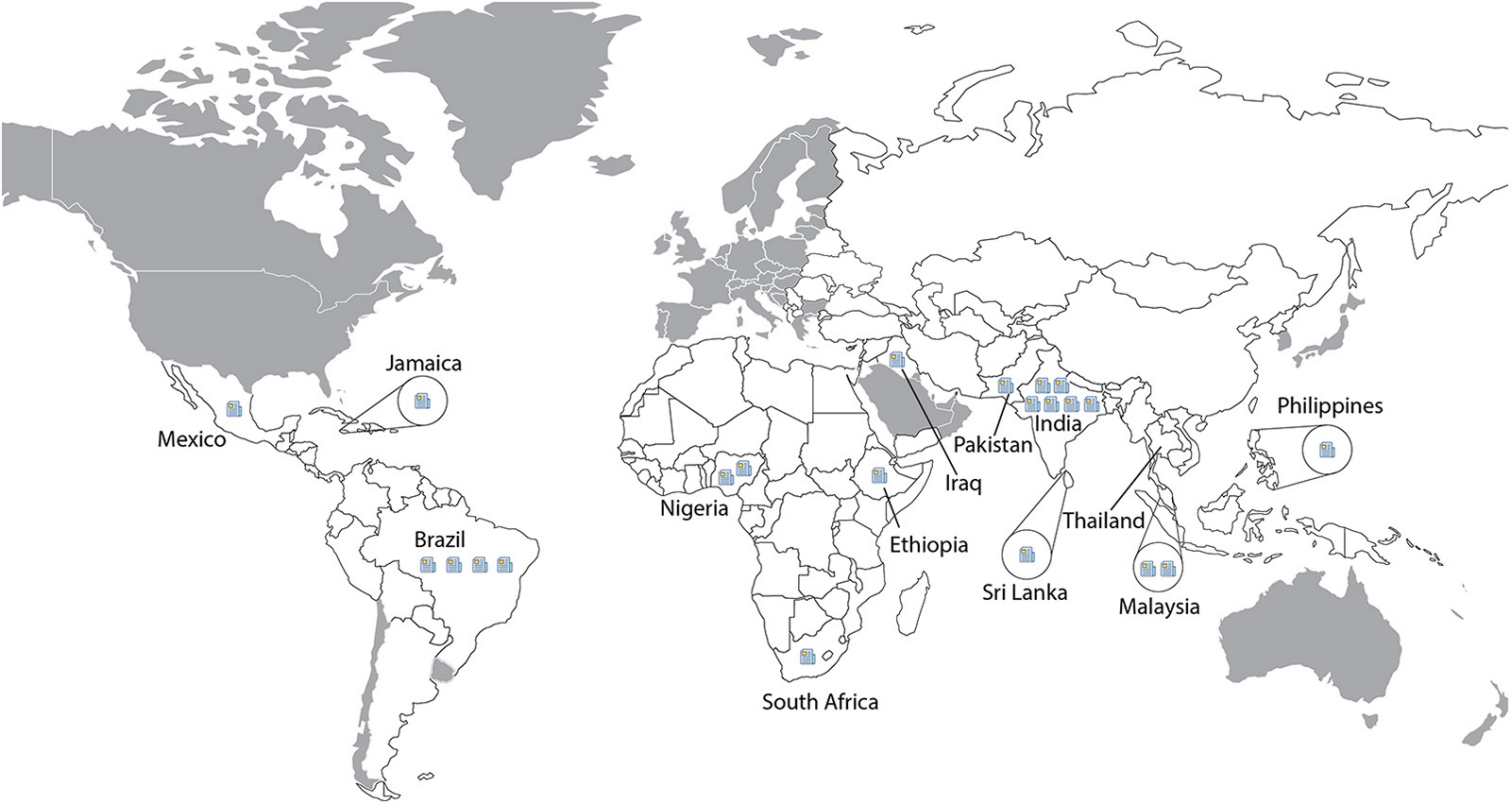
Locations of Studies on Footwear Worn by People With Diabetes In Low- and Middle-Income Countries

Only 13 countries are represented among included articles. A majority of the publications (n=14) come from South and Southeast Asia, followed by Latin America (n=5), sub-Saharan Africa (n=4), the Middle East (n=1), and the Caribbean (n=1). Four of the 5 Latin American studies are from Brazil, and 2 of the 4 studies from sub-Saharan Africa are by the same first author in Nigeria. India had the greatest number of studies (n=6). Figure 2 depicts study origins and highlights the concerning paucity of data from most world regions.

Most studies were of a quantitative, cross-sectional design, usually based in or recruiting patients from 1 or more health care facilities. Nine were published between 2010–2015, and 16 were published between 2016–2021. Twelve studies aimed to evaluate foot self-care knowledge and/or practices of people with diabetes, while 7 were specifically focused on footwear, and 4 sought to identify risk factors for DFU development. Manickum et al.^5^ recently published a thorough review of the foot-related self-care knowledge and practices of people with diabetes, and there are numerous reviews of DFU risk factors, so those results will not be presented here.

Included studies had a median of 170 participants, with a range of 38–539. Women are over-represented in many of the samples. Mean sample ages, where reported, were between 47–65 years. Nine studies included only people with type 2 diabetes. Some also included a minority of people with type 1 diabetes, and several did not state participant diabetes types.

Footwear data were acquired primarily through questionnaires and interviews (Table 1). Hence, a majority of the footwear information is self-reported. Most researchers inquired about what type of footwear participants usually used or preferred to use, but some examined the footwear that participants wore to the interview or clinic visit where the study was conducted, making the assumption that they wore that footwear often.

**TABLE 1.** Modes of Data Collection in Included Studies on Footwear Worn by People With Diabetes in Low- and Middle-Income Countries

**Method**	**Studies, No.**
Interview	2
Investigator/interviewer-administered questionnaire	6
Participant-administered questionnaire	4
Questionnaire (unspecified)	6
Observation/exam	3
Unspecified	4

Footwear classification schemes used by included studies are shown in Table 2. One study classified individual sets of footwear in more than 1 way,^41^ while 3 studies used mutually exclusive footwear categories but allowed participants to select more than 1 type (e.g., Gayle et al.^42^ asked participants if they wore different types of shoes at any time).^42^^–^^44^ One study measured the hardness of shoe soles but did not otherwise qualitatively describe the footwear.^45^ The majority of the included studies (n=20) used 1 or more qualitative, mutually exclusive footwear categories and collected only 1 data point per participant. Three of these articles also classified footwear into appropriate/inappropriate^24^^,^^52^ or poor/fair/good/optimal.^53^ Isip et al.^24^ made this judgment based on the type of shoe as well as the fit. Six articles did not report footwear used for 100% of the sample.^46^^–^^51^

**TABLE 2.** Footwear Classification Schemes Used by Included Studies for People With Diabetes

**Classification Scheme**	**Studies, No.**
Multiple nonexclusive footwear categories	1
More than 1 qualitative, mutually exclusive categories	3
1 of a set of qualitative, mutually exclusive categories	17
1 of a set of qualitative, mutually exclusive categories, and a judgment of quality	3
Measured hardness of soles	1

### Footwear Practice

Table 3 displays the percentages of participants found to be using different types of footwear in each study, along with study characteristics and pertinent qualities of samples. The characterizations of footwear types in Table 3 are reproduced verbatim from the articles to prevent any misinterpretation or misrepresentation by the reviewers, as some of the terms are not used in the reviewers’ country.

**TABLE 3.** Characteristics and Findings of Included Studies on Footwear Worn by People With Diabetes in Low- and Middle-Income Countries

**Authors,** **Year**	**Country and Setting**	**N and Sample Characteristics**	**Results**
Bañuelos-Barrera et al. 2013^41^	Mexico,primary care center	N=87, 68% neuropathic51% with foot deformityMean 6 years of education	99% standard 82% [of footwear] in good condition 52% footwear material: leather 51% open shoes 63% flat shoe tips
Brilhante Batista et al. 2020^43^	Brazil,basic health units	N=197, 91% with income at or less than minimum wage47% with less than 5 years of education	74% open-shoe sandal type 30% soft and closed-in shoes 4.1% tight closed-in shoes 1.0% pointy shoes
Chaurasia and Valame 2017^45^	India,outpatient department of tertiary care hospital	N=350, 44% rural29% with graduate education66% neuropathic65% at moderate and 10% at high risk of DFU	65% more than 35 shore units 35% 16–35 shore units 0% 8–15 shore units (where shore units are a unit of hardness, and 8–15 was considered appropriate for footwear for people with diabetes)
Chellan et al. 2011^54^	India,podiatry division of tertiary care center	N=361, all hospitalized for DFU93% neuropathic	80% sandals 16% closed shoes 2.5% therapeutic footwear 1.4% barefoot
de Sá Policarpo et al. 2014^55^	Brazil,2 family health units	N=85, 72% with family income at or less than 2 minimum wages19% said comfortable and closed shoes ideal	87% open sandals 9.4% closed and soft 3.5% closed and tight
Gayle et al. 2012^42^	Jamaica,hospital diabetes clinic	N=72, diabetes clinic attendees81% female32% with post-secondary education	Females: 88% slippers, 85% open-toe shoes, 85% broad round-toe shoes, 62% leather shoes, 62% sneakers, 43% high-heel shoes, 36% pointed- toe shoes, 22% canvas shoes, 17% plastic shoes Males: 93% slippers, 50% open-toe shoes, 71% broad round-toe shoes, 71% leather shoes, 57% sneakers, 50% pointed toe shoes, 21% canvas shoes, 6.9% plastic shoes, 6.9% work boots
Goie and Naidoo 2016^46^	South Africa,outpatient department of diabetes clinic	N=280, 76% with altered limb sensation92% visited clinic monthly9.3% had previous DFU3.6% had previous amputation	83% sandals and flip-flops
Hirpha et al. 2020^51^	Ethiopia,outpatient department of medical center	N=370, 44% female39% illiterate53% urban31% farmers36% had previous DFU	23% sandals/slippers 28% shoes without socks
Isip et al. 2016^24^	Philippines,outpatient department of medical center	N=170, 73% female47% college educated8.8% had active DFU62% at some risk of DFU	Females: 40% sandals, 31% flip-flops, 10% slip-ons, 8.8% ballet flats, 8.8% rubber shoes/sneakers, 0.7% pointed-toe shoes, 0.7% platform shoes Males: 35% sandals, 22% flip-flops, 20% slip-ons, 13% rubber shoes, 11% boat shoes
Jain and Rajagopalan 2018^56^	India,hospital surgery department	N=38, regular foot patients at outpatient department21% female47% illiterate18% had previous amputation	32% Hawaii slippers 55% ordinary slippers/chappals 5.3% therapeutic footwear 2.6% shoes (ordinary) 2.6% sandals 2.6% no footwear
Jamani et al. 2018^44^	Malaysia,diabetes clinic	N=166, 68% unemployed75% with income less than 1,500 Ringgit42% had foot problem	69% flip-flops or thongs 47% sandals 1.2% custom-made shoes
Kosachunhanun et al. 2012^57^	Thailand,tertiary care diabetes clinic	N=438, patients visiting diabetes clinic78% at low risk of DFU3.9% had active DFU	67% slippers 8.8% low-heel shoes 3.5% sports shoes 1.4% high-heel shoes 19% others
Mustafa et al. 2017^58^	Pakistan,hospital diabetes management center	N=90, 7.8% with dull foot sensation82% received foot care information11% had previous DFU	43% soft-heel shoes 41% sandals 12% leather shoes 2.2% high-heel shoes
Oliveira Neto et al. 2017^47^	Brazil,diabetes and hypertension treatment center	N=235, patients attending health center34% with incomplete elementary schooling72% with diabetes duration for 10 years or longer38% with income less than minimum wage11% had previous DFU or amputation	59% open footwear
Prekumar et al. 2017^52^	India,health center serving urban and rural patients	66 cases and 66 controls, all cases had ulcers due to footwearcontrols had diabetes but no DFU96% neuropathic35% of cases with diabetes duration less than 5 years53% cases, 62% controls use shoes 5 hours/day or less	Cases: 32% sandals with strap and toe grip; 18% sandals with strap, toe grip, and MCR insole; 29% Hawaii beach sandals; 6.1% sandals with MCR insole, soft outsole, and adjustable front and back straps; 3.0% slip-on shoes with covered uppers; 1.5% slip-in sandals without toe grip Controls: 26% sandals with strap and toe grip; 16% sandals with strap, toe grip, and MCR insole; 47% Hawaii beach sandals; 3.0% slip-on shoes with covered uppers
Rerkasem 2011^59^	Thailand,referral hospital	N=511, 65% at low risk of DFU33% neuropathic13% had active DFU	67% Hawaii slippers 8.0% low-heel shoes 3.7% sports shoes 1.4% high-heel shoes
Ruiz Roque et al. 2017^48^	Brazil,family health unit	N=63, all insulin users68% female67% never received foot care information	70% socks and closed-toe shoes
Saber and Daoud 2018^49^	Iraq,hospital diabetes center	N=250, 71% urban31% neuropathic44% with diabetes duration less than 5 years20% had previous DFU	44% round-toe shoes 33% sandals
Saurabh et al. 2014^60^	India,rural chronic disease clinic	N=103, patients attending clinic53% found to have high diabetes awareness5.8% at high risk of DFU2.9% had active DFU	79% slippers (chappals) 16% sandals without strap 3.0% sandals with strap or floaters 0% shoes or footwear with therapeutic insole
Sriyani et al. 2013^61^	Sri Lanka,outpatient department of hospital	88 cases and 80 controls, cases had leg/foot ulcers larger than 2.5 cm^2^Controls were people with diabetes without ulcers49% cases, 25% controls with income less than Sri Lankan rupee 15,000	Cases: 75% slippers, 16% sandals, 9.2% covered shoes Controls: 55% slippers, 24% covered shoes, 21% sandals
Sukthomya et al. 2021^62^	Thailand,7 hospitals	N=539, all at moderate to high risk of DFU68% with income less than 10,000 Baht66% had loss of sensation78% visited foot clinic in the previous 6 months21% had chronic ulcer	Inside: 47% barefoot, 39% slippers or flip-flops, 6.1% closed shoes, 5.4% clog shoes, 3.2% sandals Outside: 0.7% barefoot, 43% slippers or flip-flops, 25% closed shoes, 18% clog shoes, 13% sandals
Sundram et al. 2018^53^	Malaysia,3 hospital outpatient clinics	N=174, 39% had previous DFU28% had active DFU	38% open sandals without back support 13% open sandals with forking 13% closed shoes without laces or adjustable straps 13% closed shoes with laces or adjustable straps 8.6% open sandals with back support 8.6% high-heel shoes 1.7% orthotic or custom-made shoes 3.4% other
Tagang et al. 2014^63^	Nigeria,multiple hospitals	N=156, not stated	Females: 53% slippers, 19% sandals, 15% half-shoes, 13% shoes, 0% custom-molded shoes Males: 37% slippers, 29% sandals, 17% shoes, 14% half-shoes, 1.9% sports shoes, 1.3% boots, 0% custom-molded shoes
Tagang et al. 2016^64^	Nigeria,not stated	N=156, all had previous DFU	Females: 45% slippers, 24% sandals, 18% half-shoes, 11% shoes, 1.3% sports shoes, 1.3% custom-molded shoes Males: 35% sandals, 26% slippers, 17% half-shoes, 15% shoes, 5.1% boots, 1.3% sports shoes, 1.3% custom-molded shoes
Taksande et al. 2017^50^	India,rural hospital	N=200, patients without diabetic foot, amputated food, or foot ulcersNone did daily foot self-inspection3.0% had previous foot exam by physician	85% chappals

Abbreviations: DFU, diabetic foot ulcer; MCR, microcellular rubber.

Footwear was generally classified into open-toed types and close-toed types, with most studies using different kinds and numbers of subtypes. Open-toed shoes, particularly sandals (also called “chappals”) and flip-flops (also called “slippers,” “Hawaii chappals,” “Hawaii slippers,” and “sandals with forking”), were the most common footwear in nearly all (n=20) of the included studies, in clear contrast with the recommendations of international guidelines.^15^ The popularity of sandals and flip-flops was largely consistent across samples from different world regions, with lower^41^^,^^43^^,^^56^ and higher^24^^,^^58^ education levels, lower^43^^,^^44^^,^^46^^,^^62^ and higher^55^^,^^58^ income levels, lower^47^^,^^50^^,^^57^^–^^60^ and higher^24^^,^^41^^,^^46^^,^^52^^–^^54^^,^^56^^,^^61^^,^^62^^,^^64^ DFU risk levels, and from more^24^^,^^44^^,^^46^^,^^48^^,^^49^^,^^54^^,^^56^^,^^58^ or less^41^^,^^49^^,^^50^^,^^52^^,^^55^^,^^59^^,^^60^ urban locations, though not all articles thoroughly reported all of these demographic characteristics.

Open footwear, sandals, and/or flip-flops were reportedly used at rates between 23% (in Jimma, Ethiopia^51^) and 98% (in rural India^60^), where clearly reported.^24^^,^^41^^–^^45^^,^^47^^,^^49^^,^^50^^,^^52^^–^^64^ The median is 76%, calculated using only the articles in which the number of regular open footwear users could be clearly distinguished.^24^^,^^46^^,^^47^^,^^50^^,^^52^^–^^57^^,^^59^

General closed footwear was reported to be used by between 3%–70%.^24^^,^^41^^–^^43^^,^^48^^,^^52^^–^^57^^,^^59^^,^^61^^–^^64^ The outlying 70% rate was recorded by Ruiz Roque et al.^48^ in Curitiba, Brazil, and the 2 studies reporting 3% are both from South India.^52^^,^^56^ The median rate of closed footwear use is 16%. While Isip et al.^24^ found that 78 of 170 participants were wearing closed shoes at their interviews, 63 of those 78 were wearing shoes that fit inappropriately, either by length or by width.

Therapeutic or custom footwear was not an explicit category in most articles. In those in which it was, rates of usage ranged from 0–5.3%,^44^^,^^50^^,^^53^^,^^54^^,^^56^^,^^60^^,^^63^^,^^64^ despite large proportions of a majority of these samples having indications for therapeutic footwear.^44^^,^^53^^,^^54^^,^^56^^,^^64^ Only Tagang et al. reported use of offloading half-shoes (therapeutic shoes with half of the sole removed so that only half of the foot bore weight). In their 2014 and 2016 studies, roughly 15% of participants wore half-shoes. Specific details regarding the study settings and sample demographic and disease characteristics, besides all participants having a history of DFU in the 2016 study, were not given.^63^^,^^64^

Footwear category descriptions in a few articles are not entirely clear to the authors. For example, Bañuelos-Barrera et al.^41^ characterized footwear as “standard,” “in good condition,” “footwear material: leather,” “open shoes,” and/or “flat shoe tips.” Likewise, Mustafa et al.^58^ categorized shoes into “high heel,” “sandal,” “leather shoes,” or “soft heel.” It is unclear whether shoes being described as being made of leather or having a soft heel or flat tips indicates whether the shoes are open- or close-toed. Saber and Daoud^49^ used the descriptors “round toe” and “sandals,” where, again, it is uncertain whether round-toe shoes are open or closed.

### Influences on Footwear Choices

A few studies searched for correlations between footwear choice and other demographic and medical factors. Two studies found no association between demographics and footwear,^24^^,^^42^ but 1 of the 2 noted that participants with moderate DFU risk made poorer footwear choices than those with no risk factors.^24^ Sundram et al.^53^ found that female gender, lower education, lower income, and presenting with a DFU were all correlated with inappropriate footwear use.

Several authors implicated failings by health care providers in the poor foot self-care and footwear choices of participants. A number of articles reported that many patients had not ever had their feet inspected or been informed about how to care for their feet, including by making appropriate footwear choices, by a health care professional.^24^^,^^41^^,^^42^^,^^48^^,^^50^^,^^56^^,^^60^^,^^64^ Jain and Rajagopalan^56^ pointed out that none of their participants (all of whom had just received surgical treatment for diabetic foot) were advised by their providers regarding footwear, which they characterized as negligence. Large numbers of patients in other studies were judged to have low levels of knowledge regarding diabetic foot self-care,^43^^,^^47^^,^^49^ and some authors recommend more time and energy be spent on patient education.^42^^,^^50^

Isip et al.^24^ were the only investigators to inquire about the reason that participants preferred the footwear that they did. Comfort was by far the most cited reason, with only 1.6% of females and 6.5% of males citing safety concerns. Other authors suggested explanations for participants’ practices, and these explanations were very similar across countries represented in this review. Authors in Malaysia, Thailand, the Philippines, and India pointed to hot, wet climates as a reason for subjects choosing open footwear.^24^^,^^53^^,^^59^^,^^60^ Isip et al.^24^ noted that it was a significant and unsolved challenge for doctors to “reconcile what footwear is comfortable versus what is protective for the patient’s feet.”

Cultural and religious practices involving the removal of shoes several times a day were also said to make open shoes that were easy to slip on and off attractive.^53^^,^^60^^,^^62^ Finally, poverty and cost were suggested as major drivers of footwear choice and barriers to safe footwear use by authors in India, Thailand, Malaysia, and Nigeria.^52^^,^^53^^,^^59^^,^^63^ Prekumar et al.^52^ stated that popular shoes with metal buckles were not appropriate for people with diabetes but “acceptable” because “this was the most acceptable model that even a poor person could purchase.”

## DISCUSSION

The objectives of this scoping review were to explore the extent of, summarize, and interpret worldwide data on footwear usage by people with diabetes in LMICs. To this end, 25 studies from 13 countries reporting the footwear types used by samples of this population since 2010 were identified. Sandals and flip-flops were by far the most popular footwear choices.^24^^,^^41^^–^^45^^,^^47^^,^^49^^,^^50^^,^^52^^–^^64^ These choices are in clear contrast with international guidelines, which recommend close-toed footwear with thick soles and without features that can create pressure points, like straps or toe grips.^15^^,^^22^ Inadequate foot care education and low levels of diabetic foot care awareness, tropical weather, frequent cultural removal of shoes, and poverty were posited to be responsible for the popularity of poor footwear choices.^24^^,^^41^^–^^43^^,^^48^^,^^50^^,^^52^^,^^53^^,^^56^^,^^59^^,^^60^^,^^62^^–^^64^ Prekumar et al.’s apparent resignation to accepting suboptimal shoes with metal buckles because they were “the most acceptable model that even a poor person could purchase” frames poverty as an insurmountable barrier to proper footwear use for the poor.^52^ In light of this evidence, LMIC clinicians and public health programs relying on international guidelines may benefit from questioning whether the recommendations optimize health benefits in their settings given potentially low uptake. Alternative strategies that better fit LMIC contexts may be worth considering.

Sandals and flip-flops were the most popular footwear choices, in contrast with international guidelines that recommend close-toed footwear with thick soles and without straps or toe-grips that can create pressure points.

Unfortunately, few articles representing limited geographies were found by our thorough search of the literature. Many studies assessing diabetic foot self-care knowledge or practice did not include footwear practice despite the significance of footwear in DFU prevention; few of the 58 studies on this topic reviewed by Manickum et al.^5^ were eligible for inclusion in our review. Choice of footwear is a leading cause of ulceration^19^ and is, in our opinion, at least as important a self-care behavior as inspecting footwear; washing, drying, and moisturizing the feet; and other commonly reported practices. We recommend that details of footwear practice be included in these types of studies in the future, especially those conducted in LMICs, to paint a fuller picture of foot self-care practices of people with diabetes and controllable DFU risk factors.

The amount and quality of currently available data are insufficient to draw broad conclusions about all LMICs. The included studies appeared to be of generally fair methodological quality with respect to their collection and reporting of footwear data (though these data were not the primary focus of most of them). The convenience sampling at health care facilities and self-reports or 1-time observations that many of the studies used have limitations (e.g., not representative of people with diabetes not seeking health care or desirability bias), but these methods were practical given the studies’ objectives. A few articles’ reporting was incomplete, with study setting, sample characteristics, or details of data collection instruments not stated.^49^^,^^56^^,^^57^^,^^61^^,^^63^^,^^64^ Despite the relatively small number of included studies and their mixed methodological quality, the consistently high rates of open footwear use reported is cause for concern. Additionally, several other studies from LMICs classifying footwear of people with diabetes as appropriate or inappropriate but that do not define these terms or classify more than half of their sample (and hence, were not eligible for inclusion in our study) corroborate the low rates of appropriate footwear use found in included studies.^65^^–^^74^ More studies are warranted, particularly in Latin America, sub-Saharan Africa, the Middle East, Central Asia, and Eastern Europe. Little is reported in the literature about the footwear practices of people with diabetes in these regions. While local clinicians and public health officials are likely aware of common practices in their areas, reporting this information is critical. Public reports can help draw greater international attention to the issue of low rates of recommended footwear usage, spark increased global collaboration on new interventions, and reshape the international diabetic foot conversation and research agenda.

The results of this scoping review indicate that such new research and public health interventions are needed. Improper footwear is implicated in the pathogenesis of a large proportion of DFUs, which represent a serious, large-scale public health problem resulting in the loss of 1 million limbs per year and massive human and economic costs.^7^ As argued in a few little-acknowledged works, so-called “international consensus” guidelines do not appear to be serving health care or public health practitioners in LMICs, where a majority of people with diabetes live today.^23^^,^^24^^,^^75^ This issue has yet to be a focus of international interest groups like the International Diabetes Federation and International Working Group on the Diabetic Foot. Without contextually sensible guidance available to them, public health professionals and care providers are left without any actionable, evidence-based programming or clinical recommendations for DFU prevention.

New, effective strategies will be best informed by thorough data on current practices, which the present review collates, as well as the factors shaping current practices. Of the studies that report on footwear usage, few inquire about the reasoning behind the footwear choices of people with diabetes. Only 1 of the 25 articles included in this review collected data from patients regarding how they make their footwear decisions.^24^ A few other authors offered observations about patients’ physical and cultural environments, lack of financial resources, and low awareness of risks around footwear,^24^^,^^41^^–^^43^^,^^48^^,^^50^^,^^52^^,^^53^^,^^56^^,^^59^^,^^60^^,^^62^^–^^64^ but it is crucial to understand directly from patients what influences these choices and how much, so that interventions may be tailored to efficiently target the most important factors. This is especially critical where resources are limited and not all potentially promising programs can be pursued.

Seid and Tsige^76^ provided a few clues about common barriers to diabetic foot self-care in general, collected from 313 people with diabetes in Ethiopia. Obstacles cited by participants included inadequate patient-provider communication, not knowing how to care for one’s feet, inconvenience, and lack of understanding of the importance of foot care and motivation to perform foot self-care. A qualitative review also highlighted preferences for traditional and herbal medicine, underestimation of the vulnerability of the feet, and low self-efficacy in terms of being able to keep one’s feet healthy as reasons for insufficient foot self-care.^77^

Future research and public health outreach should focus on not only current footwear practice but also the reasons behind it so that public health departments can make informed decisions about, for example, whether their limited budget is better spent either subsidizing protective footwear or training primary care providers on patient foot care education. A scoping review by Paton et al.^25^ concluded that education paired with either persuasive techniques or lowering barriers to self-care were the most promising types of interventions to improve self-care of the diabetic foot, but a majority of the included studies came from North America and Europe. More data should be collected from LMICs in diverse world regions to determine what will be most effective in different contexts.

A few strategies for improving diabetic foot care have been proposed by researchers in LMICs thus far. Abbas and Archibald^75^ described their efforts to shift some tasks from doctors to less educated workers, who they trained on diabetic foot care provision. Abbas et al. and Pendsey and Abbas also reported on the success of a “train the trainers” model program implemented in Tanzania and South Asia (and since replicated at many other sites around the world), in which health care providers attended short, centralized diabetic foot care trainings and then went on to share their learnings with other providers.^31^^,^^78^^,^^79^ Jain and Apoorva^23^ proposed a footwear “ladder,” whereby physicians gradually transitioned patients from no or typical footwear to the most acceptable, “simple” therapeutic footwear (e.g., microcellular rubber sandals), and later on to more specialized shoes. Tagang et al.^63^ set out to design user-centered diabetic shoes for Nigerian patients and concluded that sandals were the most suitable type of shoe, despite acknowledging that closed footwear is recommended. They chose sandals instead because of the balance between cost, ventilation, comfort, and protective potential. Earlier work on footwear design for people with leprosy in LMICs may be useful to inform priorities in new footwear development in some cultures (e.g., Kulkarni et al. highlight the importance of discreet therapeutic features for avoiding social stigma).^80^

In addition to our findings, these works raise questions about whether the definition of “appropriate” footwear, as set by dominant Global North thinking, can or should be applied in LMICs. While rooted in medical evidence, this definition may be overly narrow, neglecting the variation in weather, socioeconomic, and cultural conditions around the world and doing a disservice to contexts that were not in focus during the formulation of the definition. Isip et al.’s observation that most of the closed shoes that patients wore were potentially harmful because of incorrect sizing^24^ further calls into question the validity of the closed footwear (good)/open footwear (bad) dichotomy used by many researchers and public health organizations, including those in LMICs and reviewed here.

Rigid, top-down DFU prevention policies are unlikely to be effective in contexts for which they were not designed. We invite diabetic foot experts and public health workers to consider a more holistic, local determination of what is and is not appropriate in terms of footwear and other diabetic foot care practices. This approach may lead to the ideation of new interventions with improved uptake and sustainability. International expert groups and authors of current authoritative guidelines still have valuable expertise, and we recommend that they collaborate to a greater extent with public health stakeholders from LMICs, consider resource constraints and cultural differences to offer different possible approaches in future guidelines, and empower LMIC practitioners to adapt methods or core concepts to their specific contexts, as needed.

“International consensus” guidelines do not appear to be serving health care or public health practitioners in LMICs, where a majority of people with diabetes live today.

We invite diabetic foot experts and public health workers to consider a more holistic, local determination of what is and is not appropriate in terms of footwear and other diabetic foot care practices.

### Limitations

This scoping review utilized a rigorous methodology to explore and summarize a novel topic but was limited by the quantity and quality of included data. A strength of the review was the inclusion of articles in any language, though still only 25 studies with limited geographical representation were identified. More than half of included studies are from South or Southeast Asia, and we hope to see more studies from a greater diversity of world regions in the future. Gray literature was excluded, which could have caused some insights to be missed, but we believe that our inclusion criteria fit the aim of the review. The quality of included sources was not formally evaluated, though all were peer-reviewed. Additionally, the footwear data that we were interested in is a relatively simple behavioral variable to measure, unlike the effectiveness of an intervention.

All included studies recruited their participants from health care settings, so people with diabetes not seeking health care were not represented in any samples. There may be significant differences in diabetic foot self-care behaviors between people with diabetes who are more or less likely to seek health care, interface with health care providers, and receive treatment for diabetes. Rural populations are also underrepresented in the literature. While recruiting from urban health care centers is convenient, we hope to see greater representation of different geographies and care-seeking behaviors in future work to more completely inform new interventions.

Most of the eligible footwear data was self-reported by study participants. Self-reported data are subject to desirability bias, though the low levels of footwear-related knowledge found in several studies would suggest a low level of susceptibility to desirability bias for many participants.^43^^,^^47^^,^^49^^,^^55^ Footwear was also recorded and reported heterogeneously in the literature. Some of the descriptions used in the included articles were not entirely clear, limiting our ability to analyze the data. However, we reproduced study results verbatim in Table 3. Terms are expected to vary around the world, so we suggest that authors aim to be as descriptive and detailed as reasonably possible for clarity among an international audience.

## CONCLUSION

The available literature points to low rates of protective footwear use among people with diabetes living in LMICs. Most of the studies included in this scoping review found that a majority of participants routinely wear sandals or flip-flops. Current international guidelines on DFU prevention are challenging to implement in low-resource settings and may have low uptake as a result. New or revised public health strategies are thus needed to help health workers in LMICs best protect the feet of patients within the constraints of their environments. Further research on footwear practice and influences shaping footwear practice, as well as the voices of public health stakeholders in LMICs, should inform the development of alternative recommendations and novel interventions to reduce the burden of preventable DFUs.

## Supplementary Material

22-00392-Reddie-Supplement.pdf

## Notes

**Peer Reviewed**

**First published online:** April 20, 2023.

## References

[1] International Diabetes Federation (IDF). *International Diabetes Federation Diabetes Atlas 10th edition*. Diabetes Atlas; 2021. Accessed April 3, 2023. https://diabetesatlas.org/atlas/tenth-edition/

[2] Armstrong DG, Swerdlow MA, Armstrong AA, Conte MS, Padula WV, Bus SA. Five year mortality and direct costs of care for people with diabetic foot complications are comparable to cancer. J Foot Ankle Res. 2020;13(1):16. 10.1186/s13047-020-00383-2. 32209136 PMC7092527

[3] Volmer-Thole M, Lobmann R. Neuropathy and diabetic foot syndrome. Int J Mol Sci. 2016;17(6):917. 10.3390/ijms17060917. 27294922 PMC4926450

[4] Lazzarini PA, Pacella RE, Armstrong DG, van Netten JJ. Diabetes-related lower-extremity complications are a leading cause of the global burden of disability. Diabet Med. 2018;35(9):1297–1299. 10.1111/dme.13680. 29791033

[5] Manickum P, Mashamba-Thompson T, Naidoo R, Ramklass S, Madiba T. Knowledge and practice of diabetic foot care – A scoping review. Diabetes Metab Syndr. 2021;15(3):783–793. 10.1016/j.dsx.2021.03.030. 33838615

[6] Armstrong DG, Boulton AJM, Bus SA. Diabetic foot ulcers and their recurrence. N Engl J Med. 2017;376(24):2367–2375. 10.1056/NEJMra1615439. 28614678

[7] International Diabetes Federation, International Working Group on the Diabetic Foot. *Diabetes and Foot Care: Time to Act*. World Diabetes Foundation; 2005. Accessed April 3, 2023. https://www.worlddiabetesfoundation.org/sites/default/files/Diabetes%20and%20Foot%20care_Time%20to%20act.pdf

[8] Shahi SK, Kumar A, Kumar S, Singh SK, Gupta SK, Singh TB. Prevalence of diabetic foot ulcer and associated risk factors in diabetic patients from North India. J Diabetic Foot Complications. 2012;4(3):83–91.

[9] Amoah VMK, Anokye R, Acheampong E, Dadson HR, Osei M, Nadutey A. The experiences of people with diabetes-related lower limb amputation at the Komfo Anokye Teaching Hospital (KATH) in Ghana. BMC Res Notes. 2018;11(1):66. 10.1186/s13104-018-3176-1. 29361966 PMC5781296

[10] Fortington LV, Geertzen JHB, van Netten JJ, Postema K, Rommers GM, Dijkstra PU. Short and long term mortality rates after a lower limb amputation. Eur J Vasc Endovasc Surg. 2013;46(1):124–131. 10.1016/j.ejvs.2013.03.024. 23628328

[11] Rathnayake A, Saboo A, Malabu UH, Falhammar H. Lower extremity amputations and long-term outcomes in diabetic foot ulcers: a systematic review. World J Diabetes. 2020;11(9):391–399. 10.4239/wjd.v11.i9.391. 32994867 PMC7503503

[12] Ogunlesi F. Challenges of caring for diabetic foot ulcers in resource-poor settings. Internet J Adv Nurs Pract. 2008;10(2).

[13] Sexton S. *Rehabilitation of People With Physical Disabilities in Developing Countries*. International Society for Prosthetics and Orthotics; 2016. Accessed April 3, 2023. https://www.ispoint.org/wp-content/uploads/2022/02/rehabilitation_of_people_wit-1.pdf

[14] Pecoraro RE, Reiber GE, Burgess EM. Pathways to diabetic limb amputation. Basis for prevention. Diabetes Care. 1990;13(5):513–521. 10.2337/diacare.13.5.513. 2351029

[15] Schaper NC, van Netten JJ, Apelqvist J, Bus SA, Hinchliffe RJ, Lipsky BA. *International Working Group on the Diabetic Foot Guidelines on the Prevention and Management of Diabetic Foot Disease*. International Working Group on the Diabetic Foot; 2019. Accessed April 3, 2023. https://iwgdfguidelines.org/wp-content/uploads/2019/05/IWGDF-Guidelines-2019.pdf

[16] Bartus CL, Margolis DJ. Reducing the incidence of foot ulceration and amputation in diabetes. Curr Diab Rep. 2004;4(6):413–418. 10.1007/s11892-004-0049-x. 15539004

[17] Bus SA, Armstrong DG, Gooday C, et al.; International Working Group on the Diabetic Foot (IWGDF). Guidelines on offloading foot ulcers in persons with diabetes (IWGDF 2019 update). Diabetes Metab Res Rev. 2020;36(Suppl 1):e3274. 10.1002/dmrr.3274. 32176441

[18] Barwick AL, Hurn SE, van Netten JJ, Reed LF, Lazzarini PA. Factors associated with wearing inadequate outdoor footwear in populations at risk of foot ulceration: a cross-sectional study. PLoS One. 2019;14(2):e0211140. 10.1371/journal.pone.0211140. 30789920 PMC6383933

[19] Lavery LA, Peters EJG, Armstrong DG. What are the most effective interventions in preventing diabetic foot ulcers? Int Wound J. 2008;5(3):425–433. 10.1111/j.1742-481X.2007.00378.x. 18593392 PMC7951312

[20] Frykberg RG, Zgonis T, Armstrong DG, et al.; American College of Foot and Ankle Surgeons. Diabetic foot disorders. A clinical practice guideline (2006 revision). J Foot Ankle Surg. 2006;45(5 Suppl):S1–S66. 10.1016/S1067-2516(07)60001-5. 17280936

[21] Apelqvist J, Bakker K, van Houtum WH, Schaper NC. Practical guidelines on the management and prevention of the diabetic foot. Diabetes Metab Res Rev. 2008;24(Suppl1):S181–S187. 10.1002/dmrr.848. 18442189

[22] Bus SA, Armstrong DG, van Deursen RW, Lewis J, Caravaggi CF, Cavanagh PR. *International Working Group on the Diabetic Foot Guidance on Footwear and Offloading Interventions to Prevent and Heal Foot Ulcers in Patients With Diabetes*. International Working Group on the Diabetic Foot; 2015. Accessed April 3, 2023. https://iwgdfguidelines.org/wp-content/uploads/2017/10/website_footwearoffloading.pdf 10.1002/dmrr.2697 26813614

[23] Jain AKC, Apoorva HC. Footwear problems in developing countries: a practical approach. Diabetic Foot J. 2021;24(1):20–25. Accessed April 3, 2023. https://diabetesonthenet.com/wp-content/uploads/pdf/dotn885896e1be5f91e0cbf4bb612f331fb2.pdf

[24] Isip JDJ, de Guzman M, Ebison AJ Jr, Narvacan-Montano C. Footwear appropriateness, preferences and foot ulcer risk among adult diabetics at Makati Medical Center Outpatient Department. J ASEAN Fed Endocrine Soc. 2016;31(1):37–43. 10.15605/jafes.031.01.07

[25] Paton J, Abey S, Hendy P, Williams J, Collings R, Callaghan L. Behaviour change approaches for individuals with diabetes to improve foot self-management: a scoping review. J Foot Ankle Res. 2021;14(1):1. 10.1186/s13047-020-00440-w. 33407755 PMC7788877

[26] Jarl G, Lundqvist LO. Adherence to wearing therapeutic shoes among people with diabetes: a systematic review and reflections. Patient Prefer Adherence. 2016;10:1521–1528. 10.2147/PPA.S112275. 27540284 PMC4982499

[27] Jarl G, Tranberg R, Johansson U, Alnemo J, Lundqvist LO. Predictors of adherence to wearing therapeutic footwear among people with diabetes. J Foot Ankle Res. 2020;13(1):45. 10.1186/s13047-020-00413-z. 32660610 PMC7359292

[28] Ehrmann D, Spengler M, Jahn M, et al. Adherence over time: the course of adherence to customized diabetic insoles as objectively assessed by a temperature sensor. J Diabetes Sci Technol. 2018;12(3):695–700. 10.1177/1932296817747618. 29281893 PMC6154238

[29] Waaijman R, Keukenkamp R, de Haart M, Polomski WP, Nollet F, Bus SA. Adherence to wearing prescription custom-made footwear in patients with diabetes at high risk for plantar foot ulceration. Diabetes Care. 2013;36(6):1613–1618. 10.2337/dc12-1330. 23321218 PMC3661819

[30] Tan S, Horobin H, Tunprasert T. The lived experience of people with diabetes using off-the-shelf prescription footwear in Singapore: a qualitative study using interpretative phenomenological analysis. J Foot Ankle Res. 2019;12(1):19. 10.1186/s13047-019-0329-y. 30949242 PMC6429698

[31] Abbas ZG. Reducing diabetic limb amputations in developing countries. Expert Rev Endocrinol Metab. 2015;10(4):425–434. 10.1586/17446651.2015.1058151. 30293495

[32] Arksey H, O’Malley L. Scoping studies: towards a methodological framework. Int J Soc Res Methodol. 2005;8(1):19–32. 10.1080/1364557032000119616

[33] Peters MDJ, Marnie C, Tricco AC, et al. Updated methodological guidance for the conduct of scoping reviews. JBI Evidence Synthesis. 2020;18(10):2119–2126. 10.11124/JBIES-20-00167. 33038124

[34] Peters MDJ, Godfrey C, McInerney P, Munn Z, Tricco AC, Khalil H. Scoping reviews. In: Aromataris E, Munn ZE, eds. JBI Manual for Evidence Synthesis. JBI; 2020. Accessed April 7, 2023. https://jbi-global-wiki.refined.site/space/MANUAL/4687342/Chapter+11%3A+Scoping+reviews

[35] Levac D, Colquhoun H, O’Brien KK. Scoping studies: advancing the methodology. Implement Sci. 2010;5(1):69. 10.1186/1748-5908-5-69. 20854677 PMC2954944

[36] Tricco AC, Lillie E, Zarin W, et al. PRISMA extension for scoping reviews (PRISMA-ScR): checklist and explanation. Ann Intern Med. 2018;169(7):467–473. 10.7326/M18-0850. 30178033

[37] Low & middle income. The World Bank. Accessed April 3, 2023. https://data.worldbank.org/country/XO

[38] Azinge N, Anizor CO. Foot-care practices among diabetics seen in a tertiary hospital in South-south Nigeria. Nigerian J Gen Pract. 2014;12(1).

[39] Hasanah U, Yusuf S, Rachmawaty R, Mukhtar M, Sandi S. Differences in foot care practice between participants at risk for and with diabetic foot ulcers (DFUs) in community. Enferm Clin. 2020;30(Suppl 2):144–148. 10.1016/j.enfcli.2019.10.010

[40] Munn Z, Peters MDJ, Stern C, Tufanaru C, McArthur A, Aromataris E. Systematic review or scoping review? Guidance for authors when choosing between a systematic or scoping review approach. BMC Med Res Methodol. 2018;18(1):143. 10.1186/s12874-018-0611-x. 30453902 PMC6245623

[41] Bañuelos-Barrera P, Arias Merino ED, Bañuelos-Barrera Y. Risk factors of foot ulceration in patients with diabetes mellitus type 2. Invest Educ Enferm. 2013;31(3):442–449.

[42] Gayle KAT, Tulloch-Reid MK, Younger NO, et al. Foot care and footwear practices among patients attending a specialist diabetes clinic in Jamaica. Clin Pract. 2012;2(4):e85. 10.4081/cp.2012.e85. 24765484 PMC3981191

[43] Batista IB, Pascoal LM, Gontijo PVC, et al. Association between knowledge and adherence to foot self-care practices performed by diabetics. Rev Bras Enferm. 2020;73(5):e20190430. 10.1590/0034-7167-2019-0430. 32638922

[44] Jamani NA, Muhammad NA, Jaffar A, Ahmad S, Tohit N. Foot problem and foot care practices among diabetic patients in a primary care clinic, Kuala Lumpur. Int J Allied Health Sci. 2018;2(3):432–444.

[45] Chaurasia A, Valame S. Diabetic foot risk assessment and foot care among patients attending tertiary care hospital in Central India. J Evol Med Dental Sci. 2017;6(72):5153–5158. 10.14260/Jemds/2017/1119

[46] Goie TT, Naidoo M. Awareness of diabetic foot disease amongst patients with type 2 diabetes mellitus attending the chronic outpatients department at a regional hospital in Durban, South Africa. Afr J Prim Health Care Fam Med. 2016;8(1):e1–e8. 10.4102/phcfm.v8i1.1170. 28155315 PMC5125263

[47] Oliveira Neto M, Pereira MDS, Pinto MAH, Agostinho LM, Reinaldo Júnior FE, Hissa MN. Evaluation of self-care for diabetic foot prevention and clinical examination of the feet in a diabetes mellitus reference center. Article in Portuguese and English. J Health Biol Sci. 2017;5(3):265–271. 10.12662/2317-3076jhbs.v5i3.1092.p265-271.2017

[48] Ruiz Roque A, Cauduro FLF, Moraes DCN. Lower limb self-care among diabetic insulin users. Fisioter Mov. 2017;30(4):813–819. 10.1590/1980-5918.030.004.ao17

[49] Saber H, Daoud A. Knowledge and practice about the foot care and the prevalence of the neuropathy among a sample of type 2 diabetic patients in Erbil, Iraq. J Family Med Prim Care. 2018;7(5):967–974. 10.4103/jfmpc.jfmpc_163_18. 30598942 PMC6259530

[50] Taksande B, Thote M, Jajoo UN. Knowledge, attitude, and practice of foot care in patients with diabetes at central rural India. J Family Med Prim Care. 2017;6(2):284–287. 10.4103/2249-4863.219994. 29302533 PMC5749072

[51] Hirpha N, Tatiparthi R, Mulugeta T. Diabetic foot self-care practices among adult diabetic patients: a descriptive cross-sectional study. Diabetes Metab Syndr Obes. 2020;13:4779–4786. 10.2147/DMSO.S285929. 33304103 PMC7723031

[52] Premkumar R, Rajan P, Rima J, Richard J. Footwear in the causation and prevention of foot ulcers in diabetes mellitus. Natl Med J India. 2017;30(5):255–261. 10.4103/0970-258X.234391. 29916424

[53] Sundram ER, Sidek MY, Yew TS. Types and grades of footwear and factors associated with poor footwear choice among diabetic patients in Usm Hospital. Int J Public Health Clin Sci. 2018;5(2):135–143.

[54] Chellan G, Srikumar S, Varma AK, et al. Foot care practice – The key to prevent diabetic foot ulcers in India. Foot. 2012;22(4):298–302. 10.1016/j.foot.2012.08.007. 22999359

[55] de Sá Policarpo N, Moura JRA, Melo Júnior EB, Almeida PC, Macêdo SF, Silva ARV. Knowledge, attitudes and practices for the prevention of diabetic foot. Rev Gaúcha Enferm. 2014;35(3):36–42. 10.1590/1983-1447.2014.03.45187. 25474838

[56] Jain AKC, S R. A prospective study of analyzing foot wear practice in patients with diabetic foot problems. Int Surg J. 2018;5(8):2818–2826. 10.18203/2349-2902.isj20183198

[57] Kosachunhanun N, Tongprasert S, Rerkasem K. Diabetic foot problems in tertiary care diabetic clinic in Thailand. Int J Low Extrem Wounds. 2012;11(2):124–127. 10.1177/1534734612446967. 22553278

[58] Mustafa A, Iqbal M, Akhtar Parvez M. Assessment of knowledge, attitude and practices of diabetics regarding their foot care. Annals Punjab Med College. 2017;11(1):43–47.

[59] Rerkasem K. Seminar review: sociocultural practices and epidemiology of diabetic foot problem: lessons from a study in Chiang Mai University Hospital, Thailand. Int J Low Extrem Wounds. 2011;10(2):86–90. 10.1177/1534734611406102. 21622485

[60] Saurabh S, Sarkar S, Selvaraj K, Kar S, Kumar SG, Roy G. Effectiveness of foot care education among people with type 2 diabetes in rural Puducherry, India. Indian J Endocrinol Metab. 2014;18(1):106–110. 10.4103/2230-8210.126587. 24701439 PMC3968714

[61] Sriyani KA, Wasalathanthri S, Hettiarachchi P, Prathapan S. Predictors of diabetic foot and leg ulcers in a developing country with a rapid increase in the prevalence of diabetes mellitus. PLoS One. 2013;8(11):e80856. 10.1371/journal.pone.0080856. 24223231 PMC3819292

[62] Sukthomya S, Ehara Y, Srisawasdi G, Suwannakin A, Katsuhira J. Foot problems, footwear habits and indoor footwear design preferences of the diabetic population in Thailand. Niigata J Health Welfare. 2021;20(2):85–92.

[63] Tagang JI, Chen CC, Pei E, Higgett N. A proposed design framework for the provision of appropriate footwear for people suffering with diabetics. Nigerian J Mater Sci Eng. 2014;5(1):28–34.

[64] Tagang JI, Pei E, Chen RC, Higgett N, Ismail DL, Abdulrasheed I. Perceived role of therapeutic footwear in the prevention of diabetic foot ulcers: A survey of patients with diabetes mellitus in Kaduna State. Nigerian J Basic Clin Sci. 2016;13(2):78–84. 10.4103/0331-8540.187357

[65] Alves Tavares T, Souza Farias de Costa LJ, da Hora Sales ML, Mota de Moraes M. Risk factors for lower-extremity ulceration and amputation in patients with diabetes mellitus. Article in Portuguese, English, and Spanish. Rev Bras Promoç Saúde. 2016;29(2):278–287. 10.5020/18061230.2016.p278

[66] Rossaneis MA, Haddad MCFL, Mathias TAF, Marcon SS. Differences in foot self-care and lifestyle between men and women with diabetes mellitus. Rev Lat Am Enfermagem. 2016;24:e2761. 10.1590/1518-8345.1203.2761. 27533270 PMC4996089

[67] Saeed N, Zafar J, Atta A. Frequency of patients with diabetes taking proper foot care according to international guidelines and its impact on their foot health. J Pak Med Assoc. 2010;60(9):732–735. 21381579

[68] Ferraz Teston E, de Souza Senteio J, dos Santos Santiago Ribeiro BM, Maran E, Marcon SS. Factores de risco para ulceração no pé de indivíduos com diabetes mellitus tipo 2. Cogitare Enferm. 2017;22(4).

[69] Carlesso GP, Gonçalves MHB, Moreschi D Júnior. Evaluation of diabetic patients’ knowledge about preventive care of the diabetic foot, in Maringá, PR, Brazil. Article in Portuguese and English. J Vasc Bras. 2017;16(2):113–118. 10.1590/1677-5449.006416 29930635 PMC5915859

[70] de Souza Medeiros MV, Pereira Paixão I, Agra G, Tamar Oliveira de Sousa A, de Lourdes André Gouveia B, Lopes Costa MM. Perfil sociodemográfico, clínico e terapêutico de pacientes com risco para pé diabético. Rev Enferm UFPE On Line. 2018;10(6):2018–2028.

[71] Silva AFRD, Moura KR, Moura TVC, Oliveira ASS, Moreira TMM, Silva ARVD. Telephone intervention in self-care practices with the feet of patients with diabetes: a randomized clinical trial. Article in Portuguese and English. Rev Esc Enferm USP. 2021;55:e03737. 10.1590/s1980-220x2020047203737. 34190883

[72] Vidya KR, Balaji S, Rm S, Lohit K. Self-care practices among of diabetic patients in a rural area of Karnataka. Natl J Community Med. 2022;12(06):148–152. 10.5455/njcm.20210619120028

[73] Xu J, Wang Y, Chen Y, Cai Y, Liu M, Zhou Q. [Clinical analysis for patients with diabetic foot among multiple centers in China]. Zhong Nan Da Xue Xue Bao Yi Xue Ban. 2019;44(8):898–904. 31570677 10.11817/j.issn.1672-7347.2019.180746

[74] de Lima LJL, Lopes MR, Botelho CAL, Cecon RS. Evaluation of self-care with feet among patients with diabetes mellitus. J Vasc Bras. 2022;21:e20210011. 35251141 10.1590/1677-5449.210011 PMC8862594

[75] Abbas ZG, Archibald LK. Challenges for management of the diabetic foot in Africa: doing more with less. *Int Wound J*. 2007;4(4):305–313. 10.1111/j.1742-481X.2007.00376.x. 17961157 PMC7951481

[76] Seid A, Tsige Y. Knowledge, practice, and barriers of foot care among diabetic patients attending Felege Hiwot Referral Hospital, Bahir Dar, Northwest Ethiopia. Adv Nurs. 2015;2015. 10.1155/2015/934623

[77] Oni D. Foot self-care experiences among patients with diabetes: a systematic review of qualitative studies. Wound Manag Prev. 2020;66(4):16–25. 10.25270/wmp.2020.4.1625. 32294056

[78] Abbas ZG, Lutale JK, Bakker K, Baker N, Archibald LK. The ‘Step by Step’ Diabetic Foot Project in Tanzania: a model for improving patient outcomes in less-developed countries. Int Wound J. 2011;8(2):169–175. 10.1111/j.1742-481X.2010.00764.x. 21266010 PMC7950840

[79] Pendsey S, Abbas ZG. The step-by-step program for reducing diabetic foot problems: a model for the developing world. Curr Diab Rep. 2007;7(6):425–428. 10.1007/s11892-007-0071-x. 18255004

[80] Kulkarni VN, Antia NH, Mehta JM. Newer designs in foot-wear for leprosy patients. Indian J Lepr. 1990;62(4):483–487. 2086685

