# Supplementary material for: A Scoping Review of Footwear Worn by People With Diabetes in Low- and Middle-Income Countries: Implications for Ulcer Prevention Programs
Source: Glob Health Sci Pract. 2023 Apr 28;11(2):e2200392. doi: 10.9745/GHSP-D-22-00392 (PMC10141434; doi:10.9745/GHSP-D-22-00392)
Supplement: 22-00392-Reddie-Supplement.pdf [file 22-00392-Reddie-Supplement.pdf]

Supplement to: Reddie M, Shallal C, Frey D. A scoping review of footwear worn by people with diabetes in low- and middle-income countries: implications for ulcer prevention programs. *Glob Health Sci Pract*. 2023;11(2):e2200392. https://doi.org/10.9745/GHSP-D-22-00392

PubMed search query:

1. diabetes[All fields]
2. AND (((“footwear) OR (foot wear) OR (shoes))[All fields])
3. AND (((worn) OR (wear*) OR (wore) OR (practice*) OR (prefer*) OR (habits))[All fields])
4. AND ("2010/01/01"[Date - Publication] : "3000"[Date - Publication])
5. NOT (guid*[Title])

As in search bar: ((((diabetes) AND (footwear OR foot wear OR shoes)) AND ((worn) OR (wear*) OR (wore) OR (practice*) OR (prefer*) OR (habits))) AND (("2010/01/01"[Date - Publication] : "3000"[Date - Publication]))) NOT (guid*[Title])
